# MicroRNA-100 Reduced Fetal Bovine Muscle Satellite Cell Myogenesis and Augmented Intramuscular Lipid Deposition by Modulating IGF1R

**DOI:** 10.3390/cells11030451

**Published:** 2022-01-28

**Authors:** Bilal Ahmad Mir, Elke Albrecht, Asghar Ali, Ola Hansson, Steffen Maak

**Affiliations:** 1 Institute of Muscle Biology and Growth, Research Institute for Farm Animal Biology, 18196 Dummerstorf, Germany; maak@fbn-dummerstorf.de; 2 Department of Clinical Sciences, Lund University, 20502 Malmö, Sweden; ola.hansson@med.lu.se; 3 Institute of Genome Biology, Research Institute for Farm Animal Biology, 18196 Dummerstorf, Germany; ali@genzentrum.lmu.de; 4 Chair for Molecular Animal Breeding and Biotechnology, Gene Centre, Ludwig Maximilian University of Munich (LMU), 81377 Munich, Germany; 5 Institute for Molecular Medicine Finland (FIMM), Helsinki University, 00290 Helsinki, Finland

**Keywords:** intramuscular fat, marbling, embryonic bovine muscle satellite cells, miRNAs, *IGF1R*, fatty acids

## Abstract

Previously, microRNA-100 (miR-100) and its putative mRNA target, insulin-like growth factor receptor-1 (*IGF1R*) were identified as differentially and inversely expressed in bovine longissimus dorsi (LD) muscles with divergent intramuscular fat (IMF) content by our group. While *IGF1R* signaling is implicated in myogenesis and muscle lipid metabolism, the underlying regulatory mechanisms are poorly understood. In the present study, we aimed to investigate the regulation of *IGF1R* by miR-100 during bovine muscle satellite cell (BMSC) myogenesis and lipid deposition. MiR-100 was confirmed to target the *IGF1R* 3′-untranslated region (3′-UTR) by luciferase reporter assay. Furthermore, expression of miR-100 and *IGF1R* was reciprocal during BMSC differentiation, suggesting a crosstalk between the two. Correspondingly, miR-100 mimic (agomiR) suppressed the levels of *IGF1R*, PI3K/AKT pathway signaling, myogenic gene *MYOG*, muscle structural components MYH7 and MYH8, whereas the inhibitor (antagomiR) had no clear stimulating effects. The *IGF1R* inhibitor (BMS-754807) curtailed receptor levels and triggered atrophy in muscle myotubes but did not influence miR-100 expression. AgomiR increased oleic acid-induced lipid deposition in BMSC myotubes supporting its involvement in intramuscular fat deposition, while antagomiR had no effect. Moreover, mitochondrial beta-oxidation and long-chain fatty acid synthesis-related genes were modulated by agomiR addition. Our results demonstrate modulatory roles of miR-100 in BMSC development, lipid deposition, and metabolism and suggest a role of miR-100 in marbling characteristics of meat animals and fat oxidation in muscle.

## 1. Introduction

Skeletal muscle development (myogenesis) predominantly occurs in the fetal stage and partly in the postnatal period [1]. During embryogenesis, primary myofibers are formed by the initial phase of myogenesis while a subsequent phase enables secondary fiber production, thus generating most of the skeletal muscle [2,3]. Although muscle growth occurs mainly through myogenesis, it also requires fibrogenesis and adipogenesis. This is because during embryonic muscle development, a greater number of mesenchymal stem cells in the mesoderm commit to myogenic fate, while a marginal percentage accounts for pre-adipogenic cells followed by differentiation into adipocytes, hence, establishing sites for intramuscular fat (IMF) accumulation [4,5,6]. Intramuscular fat can accumulate inside muscle fibers as lipid droplets (intramyocellular triacylglycerols) and as adipocytes within the muscle (intramuscular adipose tissue or marbling) [7]. Exposure to free fatty acids, such as oleic acid (OA), has been shown to increase accumulation of triglycerides in human [8] and C2C12 [9] myotubes. The differentiation of myoblasts and muscular fat accumulation require cooperative actions of myogenic regulatory factors, including myogenin (*MYOG*), myogenic determination factor (*MYOD*) and myogenic factor 5 (*MYF5*), and the transcription factors, which are regulated by multiple signaling pathways and associated receptors [10,11,12].

During embryonic and postnatal muscle development, insulin-like growth factor (IGF) signaling plays an important role by promoting cell proliferation, differentiation, development, and protein turnover [13,14]. These effects are mediated by IGF ligand binding to and auto-phosphorylation of the IGF1-receptor (IGF1R), resulting in the activation of intracellular signal transduction pathways, i.e., phosphatidylinositol 3-kinase (PI3K)/AKT pathways [14,15,16]. The PI3K/AKT-mediated effect of IGF1R is central to skeletal muscle growth and IMF deposition [15,17,18]. Hence, any dysregulation in IGF1R signaling may result in impaired muscle and IMF development. This is evidenced by the fact that the deletion of *IGF1R* in rodents causes atrophy and reduces muscle growth [19,20,21], while overexpression results in muscle hypertrophy [22,23]. Furthermore, conditional deletion of *IGF1R* in epigonadal fat pads of mice increases adipose tissue mass by predominantly promoting lipid accumulation [24]. Additionally, knockdown of both the insulin receptor and the *IGF1R* in lipodystrophic mouse muscle increases intracellular triacylglycerol accumulation [25]. Taken together, these findings indicate that appropriate regulation of IGF1R signaling is imperative for normal muscle and IMF development. However, stimulatory and regulatory mechanisms, including non-coding RNAs contributing to IGF1R signaling, during muscle and fat development remain unclear.

MicroRNAs (miRNAs) are short non-coding RNAs which suppress gene expression by complementary base pairing with the target mRNA sequence (seed sequence) usually in the 3′-untranslated region (3′ UTR) [26]. This binding results in either mRNA degradation or inhibition of translation [26]. In the last two decades, a huge number of miRNAs were identified to regulate biological processes such as embryogenesis, development, cellular proliferation and differentiation [27]. Correspondingly, miRNAs expressed in muscle and other tissues are involved in muscle growth, development and mass maintenance [28,29,30]. Interestingly, several miRNAs expressed in skeletal muscle have been implicated in IMF development, suggesting the same role for miRNAs in multiple spatiotemporal and physiologically related processes [31,32,33]. Regulation of tissue development and function by miRNAs occurs primarily through targeting signal transduction pathways, including the IGF1R/PI3K/AKT pathway [29,34,35]. IGF1R signaling is curtailed by miR-139-5p, miR-214 and miR-375 in multiple carcinoma cell types [36,37,38], miR-378 in primary cardiomyocytes [39], miR-100 in 3T3-L1 adipocytes [40], and miR-133 in murine C2C12 cell myogenesis [41], where all aforementioned miRNAs directly target and reduce *IGF1R* levels. However, miR-133a expression demonstrated a positive association with *IGF1R* in vascular smooth muscle cells [42]. Recently, a study reported *IGF1R* transcript reduction by miR-143 during primary bovine muscle cell proliferation but not in differentiation [43]. However, miRNA regulation of *IGF1R* and functional characterization in bovine muscle satellite cells is largely unknown. Antecedently, human miR-100 was reported among a set of upregulated microRNAs in skeletal muscle of patients with laminopathies [44], whereas miR-100 was downregulated in subjects suffering from a specific form of muscular dystrophy [45]. In healthy subjects, miR-100 responded to physical exercise and carbohydrate intake. The authors speculated that miR-100 and other miRNAs target genes of the PI3K-AKT pathway, ubiquitin proteasome, FOXO, and mTOR signaling pathways [46]. Moreover, significant positive correlations were found between miR-100 expression and muscle strength but not with cross-sectional muscle area [47]. These results demonstrate that miR-100 is apparently involved in human muscle growth and development in health and disease. Similarly, these effects have been shown in rodent models, whereas data in other species are scarce. In contrast, the involvement of miR-100 in buffalo adipogenesis and fatty acid composition in cattle was shown only recently [33,48]. This, together with our previous results from mRNA/miRNA profiling in bovine muscle with different IMF content motivated our current study. In our previous microarray screen, we found upregulation of miR-100 and downregulation of *IGF1R* in bovine LD muscles with high IMF content compared to low IMF content [49,50]. Whether this negative correlation exists in primary bovine muscle cells or if miR-100 plays a causal role in muscle cell proliferation and differentiation and lipid accumulation by targeting *IGF1R* remains unclear.

In this study, we investigated the regulation of *IGF1R* by miR-100 and its potential role in myogenesis and IMF accumulation in bovine muscle satellite cells (BMSC). The physical miRNA-mRNA interaction was confirmed and the modulation of IGF1R pathway and myogenic genes as well as OA deposition in myotubes under miR-100 overexpression and suppression was investigated.

## 2. Materials and Methods

### 2.1. MicroRNA Target Prediction and Dual-Luciferase Reporter Assay

We employed TargetScan7.2 (http://www.targetscan.org/vert_72/ (accessed on 20 November 2021)) [51] to determine the seed sequence for miR-100 on *IGF1R* 3′ UTR. Bovine species was chosen, and only conserved targets were selected. The oligonucleotides designed to encompass the *IGF1R* 3′UTR seed sequences for miR-100 binding site and their mutated equivalents are listed in Table 1. C2C12 myoblasts (2 × 10^5^), (ATCC^®^ CRL1772™, LGC Standards, Wesel, Germany) were seeded in 96-well white-walled plates. Twenty-four hours after seeding, cells were co-transfected with 150 ng of pmirGLO Dual-Luciferase miRNA target expression vector (Promega, Walldorf, Germany) containing either the putative miR-100-*IGF1R* binding site or its mutant control, cloned between PmeI and XbaI downstream of the Nanoluc luciferase; together with 20 nM miR-100 mimic (agomiR) (mirVana^TM^ miRNA mimic, Life Technologies, Darmstadt, Germany), or a scrambled miRNA negative control (NC) (Life Technologies, Darmstadt, Germany), using Lipofectamine 2000 (Life Technologies, Darmstadt, Germany) following the manufacturer’s protocol. Twenty-four and 48 h post-transfection, cells were sequentially assayed for Firefly and Nanoluc luciferase expression using the Nano-Glo^®^ Dual-Luciferase^®^ reporter assay kit (Promega, Walldorf, Germany) following the manufacturer’s protocol. Normalized relative luciferase activity (RLA) was calculated by the following formula: RLA = [Nluc]/[luc2 luciferase].

### 2.2. Isolation of Bovine Muscle Satellite Cells

Skeletal muscle tissues from the left foreleg of a Holstein-Friesian fetus at 4.5 months of gestation were utilized to obtain BMSC and to determine their differentiation properties. Briefly, the muscle tissue was minced in phosphate-buffered saline (PBS; 144 mM of NaCl, 5.4 mM of KCl, 25 mM of glucose, 14 mM of sucrose, 5 mM of Na_2_HPO_4_, 50 IU/mL of penicillin, 50 μg/mL of streptomycin, and 1 μg/mL of phenol red, pH adjusted to 7.4 at 22 °C). Afterward, the tissues were digested, and cells were dissociated with 0.3% trypsin (Invitrogen, Karlsruhe, Germany) in PBS for 1 h in a water bath at 37 °C with constant shaking. The 20% fetal bovine serum (FBS; Invitrogen, Karlsruhe, Germany) was used to stop digestion followed by filtering cell suspension through three layers of sterile nylon mesh (2 × 63 μm, 1 × 20 μm pore size), diluted 1:1 in PBS, and centrifuged at 250× *g*, 4 °C for 10 min. The cell pellet obtained was resuspended in PBS, and trypan blue stained cell mix was used to determine cell number with a Neubauer counting chamber (Roth, Karlsruhe, Germany). Cells were centrifuged and resuspended again followed by seeding at approximately 10^5^ cells/cm^2^ on 100-mm Primaria plastic petri dishes (Falcon, Becton Dickinson, Heidelberg, Germany) in Dulbecco’s Modified Eagle Medium (DMEM) supplemented with 0.02 M of glutamine (Serva, Heidelberg, Germany), 100 IU/mL of penicillin, 100 μg/mL of streptomycin, and 10% FBS. Cells were incubated under a humidified atmosphere of 6% CO_2_ at 37 °C. Twenty-four hours later, cells were washed with PBS, and media was replaced with fresh medium (DMEM + 10% FBS + 1% antibiotics). After 48 h, cell monolayers were collected using a trypsin/EDTA solution (0.05%/0.02%, Roth, Karlsruhe, Germany) in PBS. Cells were counted and 2-mL aliquots with 1.08 × 10^6^ cells/mL were frozen in liquid nitrogen using DMEM containing 20% FBS and 10% dimethyl sulfoxide (DMSO, Serva, Heidelberg, Germany) until required for further experiments.

### 2.3. Muscle Satellite Cell Culture and miRNA Transfection

Isolated muscle satellite cells were seeded at a density of 1 × 10^4^ cells/well in collagen-1 or Matrigel (Corning, New York, NY, USA) coated 6- and 12-well plates. The cells were grown in DMEM supplemented with 10% FBS and 100 IU/mL of penicillin-streptomycin under humidified conditions of 6% CO_2_ at 37 °C. After 3 days, when cells were approximately 90% confluent, growth media was replaced with differentiation media containing DMEM supplemented with 2% FBS and 100 IU/mL of penicillin-streptomycin (PAN-Biotech, Aidenbach, Germany). Media was changed every other day until day 5. Cells were harvested at different time points of 48 h (-2D) and 24 h (-1D) before differentiation, at differentiation start (Day 0) and after 24 h (Day 1), 72 h (Day 3) and 120 h (Day 5). MicroRNA-100 mimic (agomiR) and inhibitor (antagomiR) and their corresponding negative controls (NC, 20–50 nM), included in the assay kit MC10188 (order # 4464066, Thermo Fisher Scientific, Schwerte, Germany), were transfected using Lipofectamine 2000 (Thermo Fisher Scientific, Schwerte, Germany) and Opti-MEM I reduced serum medium at day 0 and primary myotubes collected at day 3 for further analyses. Sequences for bta-miR-100 mimic, inhibitor and NC as well as the oligo chemistry are available at Thermo Fischer Scientific [52]. To inhibit IGF1R, cells at day 0 were treated with 20 nM BMS-754807 (BMS, Hycultec, Beutelsbach, Germany) or DMSO and collected for expression analyses at day 3. For the induction of lipid accumulation, day 3 myotubes were treated with DMEM containing 2% fatty acid-free bovine serum albumin and 500 µM of oleic acid (OA, dissolved in ethanol) and analyzed after 24 h.

### 2.4. RNA Extraction and Real-Time PCR

Total RNA was isolated using QIAzol Lysis Reagent (Qiagen, Hilden, Germany) according to the manufacturer’s protocol and quantified using the NanoDrop 1000 spectrophotometer (Peqlab, Erlangen, Germany). One microgram of RNA was reverse-transcribed to generate first strand of cDNA using the High Capacity cDNA reverse transcription kit (iScript cDNA Synthesis Kit, Bio-Rad, Munich, Germany) according to manufacturer’s instructions. Semi-quantitative polymerase chain reaction (qPCR) was performed using the LightCycler^R^ 96 system (Roche, Basel, Switzerland) with SYBR Green Master Mix (Applied Biosystems, Carlsbad, CA, USA). The gene expression was measured in duplicates for each sample in 20-µL reaction volumes containing 10 ng of cDNA, 2 µM of the respective forward and reverse primers and 5 µL of SYBR Green Supermix (Bio-Rad, Munich, Germany). Primers were designed with Primer 3 web (https://primer3.ut.ee/ (accessed on 10 July 2020)) and synthesized by Sigma-Aldrich. The qPCR amplification conditions comprised an initial denaturation step (95 °C for 3 min) followed by 45 cycles (95 °C for 10 s, 60 °C for 30 s, 70 °C for 45 s). The primer sequences for various gene expression analyses are given in Table 2. The relative gene expression is expressed as fold difference, which was calculated using the 2^−ΔCt^ formula [53] following normalization to *GAPDH* cycle threshold (Ct) levels.

### 2.5. Protein Extraction and Western Blotting

Cells were washed with cold PBS and collected in CelLytic MT lyses reagent and Protease Inhibitor Cocktail (Sigma-Aldrich, Munich, Germany) to extract total protein according to manufacturer’s instructions. Cell lysates were centrifuged at 14,000 rpm for 15 min to remove insoluble compartments. The supernatant was transferred to a new reaction tube. Protein abundances were determined using a Jess Simple Western device (ProteinSimple, Bio-Techne, San Jose, CA, USA). Samples with a protein concentration of 1 mg/mL were prepared according to the manufacturer’s instructions and separated with the 12–230 kDa Separation Module (ProteinSimple, Bio-Techne, San Jose, CA, USA). The antibodies were diluted 1:50 (MYH7 and MYH8), 1:40 (AKT), 1:20 (pAKT), and 1:25 (IGF1R and MYOG). The RePlex feature and Total Protein Assay (Protein Simple, Bio-Techne, San Jose, CA, USA) were employed to determine the total protein amount within the same capillary, which was used for normalization. Data was analyzed with the Compass for Simple Western Software (v.6.0.0, Build 0408, ProteinSimple, Bio-Techne, San Jose, CA, USA).

### 2.6. Detection of miR-100 Expression

MicroRNA expression levels were measured by qPCR using specific primer-probe sets and the Taqman Universal MasterMix II, no UNG, kit as per the manufacturer’s instructions (Applied Biosystems, Carlsbad, CA, USA). Details were described previously [30]. For miRNA first-strand cDNA synthesis, total RNA (50 ng) was reverse-transcribed using the Taqman microRNA RT kit (Applied Biosystems, Carlsbad, CA, USA). A customized RT primer pool was prepared by pooling all miRNA-specific stem-loop primers. The reaction mix was reverse-transcribed by incubating for 30 min at 16 °C, 30 min at 42 °C, and 5 min at 85 °C. The qPCR conditions consisted of 1 cycle of 10 min at 95 °C, 40 cycles of 15 s at 95 °C, and 60 s at 60 °C. The TaqMan™ small nuclear RNA (snRNA) U6 control assay was used as a normalizing control. Samples were analyzed in triplicate for all miRNA targets using a LightCycler 96 real-time qPCR system (Roche, Basel, Switzerland). Quantitation cycle (Cq) value was calculated by the LightCycler 96 system software.

### 2.7. Oil Red O Staining

Bovine primary myotubes treated with OA for 24 h were stained with Oil Red O as described by Schering et al. [54]. Briefly, cells were washed twice with pre-warmed PBS and fixed with 4% paraformaldehyde (PFA, Roth, Karlsruhe, Germany) in PBS for 30 min at room temperature, followed by 5 min of incubation in 60% isopropanol. Subsequently, cells were incubated with fresh Oil Red O working solution for 5 min and immediately rinsed 3–5 times with distilled water. Wells were covered with gelatin (Waldeck, Münster, Germany) and respective cover slips (Roth, Karlsruhe, Germany). An inverse phase contrast microscope (Nikon Diaphot 300; Nikon, Düsseldorf, Germany), equipped with a CC-12 camera (OSIS, Münster, Germany), and the image analysis software Cell^F (OSIS, Münster, Germany) were used to analyze the intracellular lipid deposition. The fat particle number and distance, as well as fat area, percentage, and particle size were determined with a self-made macro program using the Cell^F software as described previously by our group [55]. For each well, 20 images were analyzed, accounting for a total area of 8.3 mm².

### 2.8. BrdU (5-bromo-2′-deoxyuridine) Labelling of Myoblasts

A 10 mM BrdU solution (ab142567; Abcam, Cambridge, UK) was prepared by mixing 3 mg of stock with 1 mL autoclaved dH_2_O. A 10-µM solution was prepared in culture medium and filtered through a 0.2-µm filter under sterile conditions. Twenty-four hours after seeding 0.5 × 10^4^ cells/well, cells were incubated at 37 °C in a CO_2_ incubator and labelled with 10 µM of BrdU labelling medium for 4 h. Afterward, cells were washed and BrdU staining performed. Three wells per treatment were prepared and analyzed. Cells were washed twice with PBS and fixed with 4% PFA for 15 min. After washing and permeabilization with PBST (PBS containing 0.1% TritonX-100, Sigma-Aldrich, Munich, Germany) for 10 min, cells were incubated with 2N HCl at 37 °C for 20 min. Three times of 5 min washing were followed by a blocking step with 10% normal goat serum (NGS) in PBST for 15 min. Incubation with the anti-BrdU antibody (Roche, Mannheim, Germany, 1:100 with 2% NGS in PBST) was carried out overnight at 4 °C in a humidity chamber. Cells were washed 3 × 10 min with PBST and were then incubated with the secondary antibody (Alexa Fluor 488 goat-anti-mouse IgG, 1:500, Thermo Fisher Scientific, Schwerte, Germany) for 45 min in the humidity chamber. Nuclei were counterstained with propidium iodide after 2 × 10 min washing with PBST and 1 × 10 min with PBS. Finally, wells were washed with PBS and dH_2_O and covered with ProLong Diamond Antifade Mountant (Thermo Fisher Scientific, Schwerte, Germany) and appropriate cover slips. Fluorescence was observed with a Nikon Microphot SA microscope (Nikon, Düsseldorf, Germany) and a CC-12 color camera (OSIS, Münster, Germany). The Cell^F image analysis software (OSIS, Münster, Germany) was used to determine the percentage of proliferating cells, by counting the number of BrdU-positive nuclei and total number of nuclei in 10 randomly selected areas, accounting for a total area of 10.7 mm² per sample.

### 2.9. Immunocytochemistry

Localization of IGF1R, MYOG, MYH7, and MYH8 was done in differentiated myogenic cells with immunocytochemistry using a similar protocol as described for BrdU detection, but without the denaturation step with HCl. Briefly, cells were washed, fixed with 4% PFA, permeabilized with PBST, blocked with 10% NGS in PBST, incubated with the primary antibody overnight at 4 °C in the humidity chamber, washed, and incubated with the secondary antibody at RT for 45 min. Finally, nuclei were counterstained with Hoechst 33258 (1:10,000) for 5 min, wells were washed with PBS and dH_2_O and covered with ProLong Diamond Antifade Mountant (Thermo Fisher Scientific, Schwerte, Germany) and appropriate cover slips. Primary antibodies were purchased by antibodies-online (IGF1R, ABIN1532901; MYH7, ABIN3043105) and Abcam (MYOG, ab1835; MYH8, ab210947) and were used 1:100 in PBST with 2% NGS. Secondary antibodies (Alexa Fluor 488 goat-anti-rabbit IgG and Alexa Fluor 594 goat-anti-mouse IgG, Thermo Fisher Scientific, Schwerte, Germany) were diluted 1:500 in PBST. The same equipment as described before was used for imaging.

### 2.10. Statistical Analyses

All data were analyzed with simple ANOVA or GLM procedure of SAS statistical software (Version 9.4, SAS Inst., Cary, NC, USA), with treatment as fixed factor, and are reported as the mean ± standard error of the mean. The Tukey test was used as the post-hoc test. Expression values are presented as fold differences of NC. Values of miR-100 expression, after transfection with agomiR or antagomiR compared to NC were log transformed before ANOVA to equalize variances among sample groups. Data were considered statistically significant if *p* < 0.05 and a trend if 0.05 < *p* < 0.1.

## 3. Results

### 3.1. MiR-100 Directly Targets Bovine IGF1R

**Figure 1:**
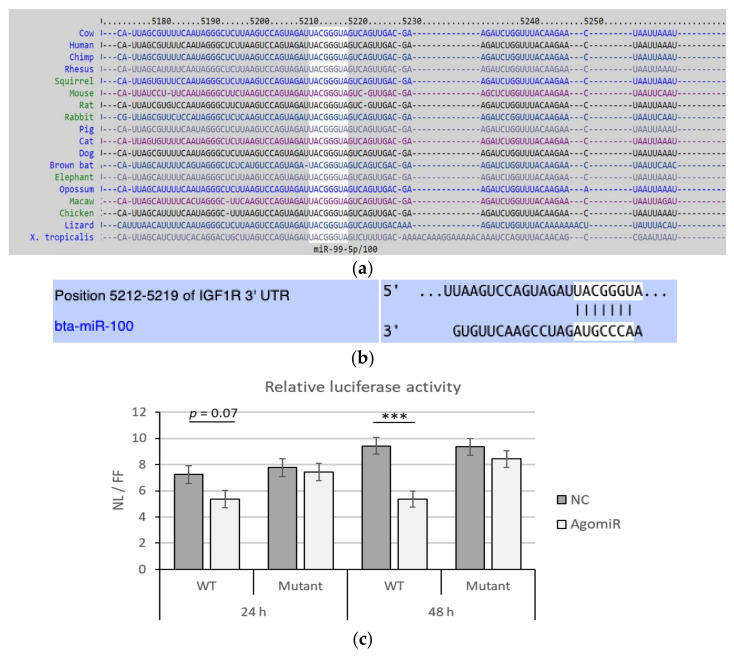
Functional miR-100 binding site identification and physical interaction on *IGF1R* 3′ UTR. Seed-matched sequences in the *IGF1R* 3′ UTR for miR-100 are conserved across species with white background in the figure, (**a**) including bovine (**b**). Dual-luciferase reporter assays determining the interaction of overexpressed miR-100 (AgomiR) and negative control (NC) with the miR-100 seed site (wildtype, WT) or its mutated version (Mutant) post 24 and 48 h of transfection (**c**). Data is representative of four independent experimental replicates per sample group. Relative expression levels (NanoLuc/Firefly-NL/FF) are presented, and if significantly different, it is indicated with *** (*p* < 0.001).

The miR-100 seed sequence for miRNA-mRNA binding in 3′-UTR of *IGF1R* is conserved across different species, including mice and humans (Figure 1a,b). To confirm whether miR-100 targets *IGF1R* by binding in its 3′-UTR, luciferase assay was performed using plasmids containing either wild-type or mutant miR-100 seed sequences. Co-transfection with wild-type seed sequence and agomiR caused 25.9% and 43.1% decrease in luciferase activity after 24 (*p* = 0.07) and 48 h (*p* < 0.001), respectively (Figure 1c). Moreover, no change in luciferase activity was observed in the cells co-transfected with plasmid containing mutated miR-100 seed sequence and agomiR (Figure 1c).

### 3.2. Correlative Expression of miR-100 and IGF1R in Bovine Muscle Satellite Cell Myogenesis

**Figure 2:**
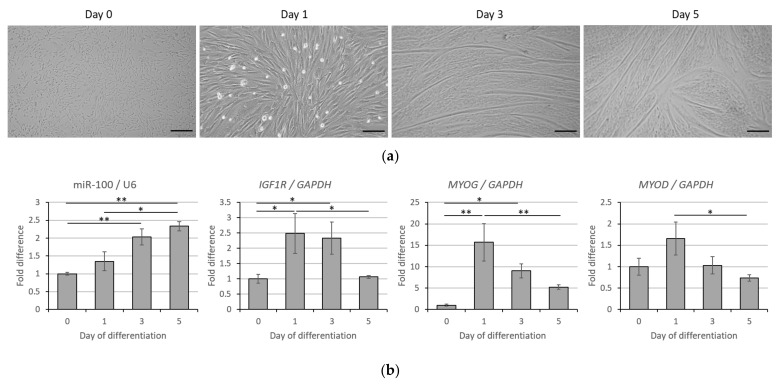
*IGF1R* and miR-100 expression during bovine muscle satellite cell differentiation. Morphology of cells on days 0, 1, 3, and 5 of myogenic differentiation (**a**) and transcript expression of miR-100 and *IGF1R* as well as of *MYOG* and *MYOD* for proper myogenic determination (**b**). Data is representative of four independent experimental replicates per sample group. Expression levels are presented as fold differences to day 0 and * and ** indicate significant differences among days with *p* < 0.05 and *p* < 0.01, respectively. Scale bar in (**a**) represents 200 µm (day 0, 3, and 5) or 100 µm (day 1), respectively.

To determine their potential role in muscle cell differentiation, the expression of miR-100 and *IGF1R* was monitored during BMSC differentiation. Microscopic visualization of BMSC and their differentiation into myotubes at different times is shown in Figure 2a. MiR-100 level increased gradually during the differentiation of satellite cells (Figure 2b, *p* < 0.05). Conversely, *IGF1R* transcript levels increased during BMSC fusion to form myocytes but declined as the cells differentiated into mature myotubes (Figure 2b, *p* < 0.05). The expression levels of *MYOG* and *MYOD* were measured to determine the correct myogenic process. The level of *MYOG* mRNA increased at day 1 (*p* < 0.01) followed by a gradual decrease at days 3 and 5 after induction of differentiation (Figure 2b, *p* < 0.05). A similar trend was seen in *MYOD* level during differentiation (Figure 2b).

### 3.3. MiR-100 Modulates IGF1R/PI3K/AKT Signaling through IGF1R Suppression

**Figure 3:**
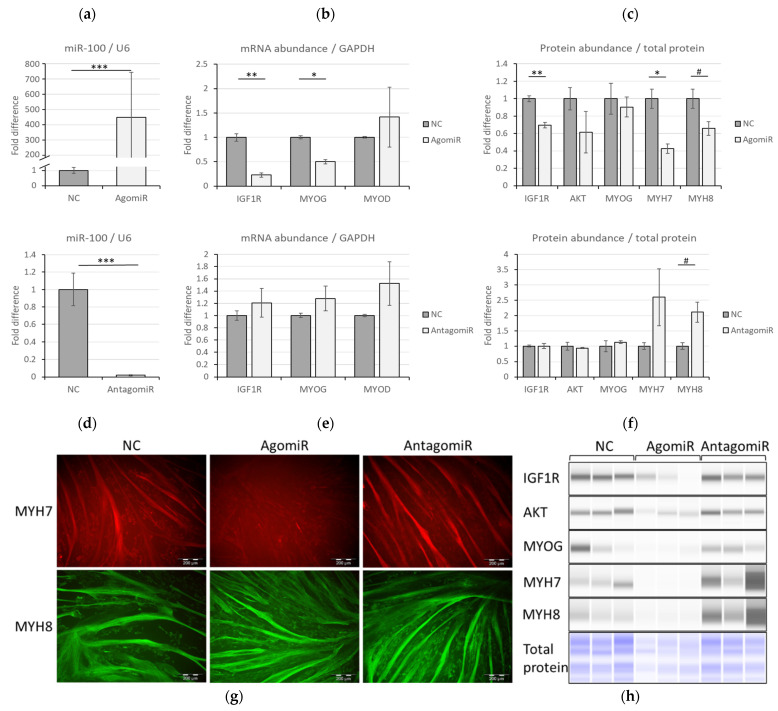
Overexpression and inhibition effects of miR-100 in myotubes. Overexpression of miR-100 in BMSC myotubes compared to negative control (NC) caused by agomiR (**a**) and the impact of this intervention on *IGF1R, MYOG,* and *MYOD* transcript levels (**b**) and protein abundances (**c**). The impact of antagomiR on miR-100 levels (**d**), *IGF1R, MYOG,* and *MYOD* levels (**e**) as well as on IGF1R, AKT, MYOG, MYH7, and MYH8 protein abundances (**f**). Protein abundances were determined through Jess Simple Western and normalized to total protein. Virtual blot images are shown (**h**), as well as immunocytochemical staining of MYH7 and MYH8 (**g**). Data is representative of three independent experimental replicates per sample group where expression levels are presented as fold differences. Significant differences to NC are indicated with * if *p* < 0.05, ** if *p* < 0.01, *** if *p* < 0.001 and a trend is indicated with # (0.05 < *p* < 0.1).

Next, we explored whether manipulating miR-100 levels would impact *IGF1R* transcript levels and myogenic differentiation. Figure 3a confirms miR-100 overexpression in BMSC myotubes 72 h after transfection with agomiR compared to NC (*p* < 0.001). AgomiR reduced the transcript levels of both *IGF1R and MYOG* (*p* < 0.05) (Figure 3b) and IGF1R protein level (*p* = 0.003) (Figure 3c). The abundance of *MYOD* mRNA (Figure 3b), and MYOG protein (Figure 3c) were not significantly influenced by miR-100 overexpression (*p* > 0.05). The level of miR-100 was significantly reduced (*p* < 0.001) upon transfecting myotubes with antagomiR compared to corresponding NC (Figure 3d). However, antagomiR did not affect the expression of *IGF1R*, *MYOG* and *MYOD* (Figure 3e; *p* > 0.16). Moreover, there was no variation in protein levels of IGF1R and MYOG (*p* > 0.05) by miR-100 inhibition (Figure 3f). Key structural proteins for muscle development, MYH7, slow twitch fibers, and MYH8 representing developing fibers, were analyzed by western blot and immunocytochemistry. In addition to reducing MYH7 (*p* = 0.02) and MYH8 (*p* = 0.07) proteins (Figure 3c), treatment of the cells with agomiR greatly reduced the number of MYH7-stained fibers (Figure 3g). This effect was reversed by the application of antagomiR (Figure 3g). In contrast, no influence was observed on MYH8 staining, but protein quantity tended to increase (*p* = 0.066) (Figure 3f,g). In addition, there was a trend to reduction of total AKT levels with agomiR, but no effect was observed with antagomiR (Figure 3f,h), while phosphorylated AKT levels were undetectable.

### 3.4. IGF1R Inhibition Has No Impact on miR-100 Levels

**Figure 4:**
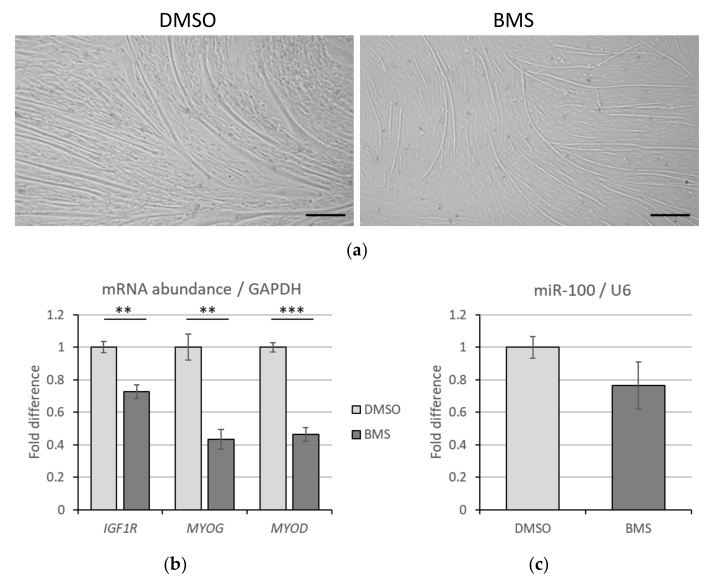
Impact of molecular inhibition of IGF1R on miR-100 levels and myogenic differentiation. (**a**) Morphology of myotubes treated with IGF1R inhibitor, BMS-754807 (BMS). Scale bar represents 200 µm. (**b**) Impact of BMS on *IGF1R*, *MYOG,* and *MYOD* transcript levels and (**c**) miR-100 expression in BMSC myotubes. Data is representative of three independent experimental replicates per sample group. Expression levels are presented as fold differences. Significant differences to DMSO are indicated with ** if *p* < 0.01 and with *** if *p* < 0.001.

To determine whether inhibiting IGF1R would affect miR-100 expression in BMSC, an inhibitor of IGF1R, BMS-754807 (BMS), was used. Addition of 20 nM of BMS for 3 days post differentiation did not affect myotube number but caused myotube atrophy compared to DMSO treated myotubes (Figure 4a), as evident through microscopic morphology. The mRNA levels of *IGF1R, MYOG,* and *MYOD* were reduced by 27%, 57%, and 53% (*p* < 0.01), respectively, by BMS compared to DMSO-treated myotubes (Figure 4b). The treatment of BMS, however, did not alter the level of miR-100 in myotubes (*p* = 0.21, Figure 4c).

### 3.5. MiR-100 Did Not Influence Proliferation of BMSC

**Figure 5:**
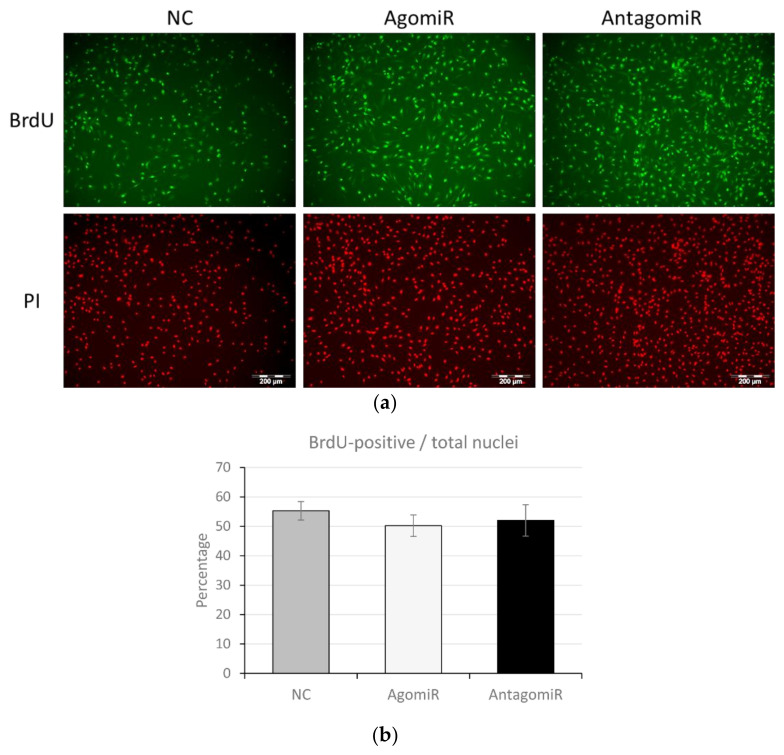
Impact of altered miR-100 levels on bovine muscle satellite cell proliferation. (**a**) Immunocytochemical detection of BrdU incorporated in nuclei of proliferating cells (green) and total nuclei stained with propidium iodide (PI, red) in cells treated with agomiR and antagomiR. Scale bar represents 200 µm. (**b**) The percentage of BrdU positive nuclei normalized to total nuclei in the well in presence of agomiR and antagomiR compared to negative control (NC). Data is representative of three independent experimental replicates per sample group.

The impact of agomiR and antagomiR on BMSC proliferation determined through BrdU incorporation and staining revealed that altered miR-100 level had no significant influence on cell proliferation (Figure 5a). The number of BrdU positive nuclei relative to total nuclei was not different in agomiR- (*p* = 0.68) or antagomiR (*p* = 0.85)-treated myoblasts compared to NC (Figure 5b).

### 3.6. Overexpression of miR-100 Promotes Oleic Acid-Induced Lipid Deposition and Reduces Lipid Oxidation in Myotubes

**Figure 6:**
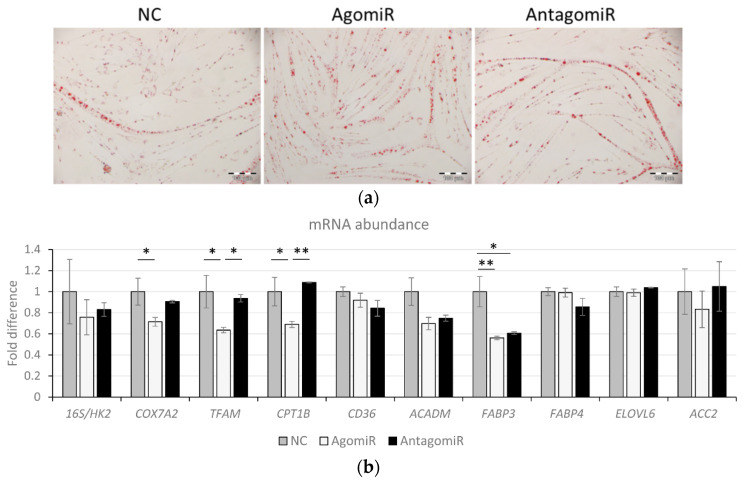
Impact of agomiR and antagomiR on oleic acid accumulation in bovine satellite cell myotubes. (**a**) Morphological images of Oil red O stained myotubes treated with agomiR, antagomiR, and negative control (NC) plus oleic acid (OA). Scale bars represent 100 µm. (**b**) The qPCR results determine transcript levels of fat oxidation and metabolism related genes in myotubes treated with OA in presence of agomiR and antagomiR. Data is representative of two or three independent experimental replicates per sample group. Expression levels were normalized to *GAPDH* and are presented as fold differences to NC. Significant differences among treatments are indicated with * if *p* < 0.05 or ** if *p* < 0.01.

In bovine LD muscles with higher IMF content, miR-100 was upregulated, suggesting that it may exert a regulatory effect on intramuscular lipid accumulation and fatty acid synthesis. To causally link this in a cell culture model, BMSC myotubes were treated with OA (500 µM) for 24 h in the presence of agomiR, antagomiR, or corresponding NC. Oil red O staining demonstrated that agomiR promoted OA-induced lipid accumulation compared to NC, as indicated by microscopic analysis (Figure 6a). Image analyses demonstrated that agomiR instigated increased fat area percentage compared to antagomiR (*p* = 0.027) and increased number of fat particles/mm^2^ compared to both other groups (*p* < 0.05) (Table 3). Interestingly, there was a trend for smaller lipid droplets in both treatment groups compared to NC (0.05 < *p* < 0.1). We next analyzed mitochondrial copy number (determined by the ratio of mtDNA (16S RNA) to the nuclear gene, hexokinase 2 (*HK2*)), markers of β-oxidation of fatty acids (*COX7A2, TFAM, CPT1B, ACAMD),* and fatty acid uptake (*CD36*) and synthesis (*ELOVL6* and *ACC2*). In addition, *FABP3* and *FABP4* were investigated as muscle fiber- and adipocyte-specific markers of triacylglycerol binding and mobilization. The agomiR significantly reduced mRNA levels of *COX7A2, TFAM*, and *CPT1B* (Figure 6b, *p* < 0.05) compared to NC, but antagomiR had no influence on these genes. The levels of *FABP3* were reduced by both agomiR and antagomiR compared to NC (Figure 6b, *p* < 0.05), while *CD36*, *ACADM*, *FABP4,* and *ELOVL6* remained unchanged in both treatments compared to NC (Figure 6b).

## 4. Discussion

In this study, we identified a distinctive causal role for miR-100 during bovine skeletal myogenesis through regulation of *IGF1R*, downstream PI3K/AKT pathway signaling, myogenic differentiation markers, and muscle structural components *MYH7* and *MYH8*. The molecular inhibition of *IGF1R* resulted in atrophied myotubes but had no influence on miR-100 levels. Moreover, we demonstrated increased OA accumulation and reduced expression of genes related to triacylglycerol oxidation by miR-100 overexpression in BMSC myotubes. These findings support positive regulation of fat accumulation by miR-100 in BMSC, suggesting its potential for intramuscular adipose tissue or marbling characteristics in meat animals.

The *IGF1R* expression and signaling is important for muscle development and is directly involved in intracellular signal transduction of hypertrophic extracellular signals in chicken myocytes [56]. Appropriate transcriptional and posttranscriptional regulation of *IGF1R* is indispensable for its typical physiological expression and function [57,58]. Here, we show reciprocal expression of miR-100 and *IGF1R* and demonstrate that repression of *IGF1R* levels by miR-100 influenced BMSC differentiation. This reduction was associated with decreased abundance in myogenic genes and AKT, resulting in blunted differentiation and protein expression signaling in muscle cells, which are central to cellular processes, such as glucose metabolism, cell proliferation, differentiation, and growth. MicroRNAs exert profound effects by cooperatively regulating multiple components in the same signaling pathway. MicroRNA-100, miR-99a, and miR-99b belong to the miR-99 family and hence, share the same seed sequence and target members of protein synthesis pathway [59]. These miR-99 family members negatively regulate IGF1R or downstream protein synthesis signaling molecules, including MTOR and RPTOR in cancer cells, macrophages, and dermal cells [59,60,61,62]. The miR-99 family members led to a reduction only in IGF1R protein level, while *IGF1R* mRNA expression remained unaffected in dermal cells [59]. Moreover, miR-99a, through myotubularin-related protein 3 inhibition, reduced myotube formation [63], while miR-99b, through RPTOR suppression, inhibited protein synthesis [64] in chicken and in human satellite cell-derived myotubes. This suppressed skeletal myotube formation is associated with reduced myogenic marker genes [63], which was also observed in this study. While miR-99a promotes proliferation of chicken muscle satellite cells [63], miR-100 had no effect on BMSC proliferation in our study although it blocks cancer cell division [65]. The muscle specific miRNAs, miR-1 [66] and miR-133 [41] repress IGF1R and associated signaling in murine C2C12 myotubes via reduction of AKT phosphorylation, i.e., an upstream target of MTOR and RPTOR. Although this study provides functional and regulational perspectives of miR-100 in BMSC differentiation through IGF1R regulation, determining its impact on the multiple downstream partners of IGF1 signaling pathway would further reveal its role in muscle cell growth. MiR-100 has been shown to regulate MTOR and IGF1R expression in 3T3-L1 adipocytes [40]. Our study demonstrates that miR-100 has the ability to inhibit *IGF1R* and thereby mediate the downstream effects. Molecular inhibition of *IGF1R* by BMS-754807, however, did not influence miR-100 levels, corroborating an upstream regulatory role of the miRNA in the IGF1R signaling cascade.

The positive role of IGF1R signaling in inducing myotube hypertrophy involves, in addition to other effects, upregulation of the structural muscle molecules MYH7 and MYH8 [15,67,68]. This signaling is disturbed in atrophy, such as in myosteatosis, in which pathologic accumulation of lipids with decreased muscle mass occurs together with downregulation of sarcomeric and structural proteins like MYH7 [69]. Likewise, perturbing the cell surface receptor, IGF1R, and thus the myogenic differentiation process, agomiR dampened MYH7 and MYH8 expression in BMSC myotubes, while antagomiR had the opposite effect in the current study. Myosin genes comprise miRNAs, such as miR-208b and miR-499, within their introns, which not only regulate myosin content and fiber identity but also regulate MYH7 and MYH7b isoform expression [70]. Our study indicates additional regulation of myosin genes by miR-100. This miRNA is not encoded within the myosin genes in contrast to miR-208b and miR-499. However, determining whether this modulation is direct or indirect needs further investigation.

Muscle is a metabolically active tissue in which lipid overflow and excess fat accumulation leads to insulin resistance [71] and impaired muscle function [72] in humans but improves marbling characteristics in meat animals [73]. As miR-100 levels were elevated in bovine LD muscles with high IMF content, we here investigated its potential causal role in fatty acid accumulation in BMSC myotubes. Moreover, because oleate can induce triacylglycerol accumulation in myotubes [74] through IGF binding protein 5 [9] and AKT-signaling, we investigated the effect of miR-100 on OA-induced lipid deposition in a BMSC in vitro model by overexpressing or inhibiting miR-100. Our results demonstrated that, in addition to reducing expression of fat oxidation-related genes, agomiR significantly promoted lipid accumulation in myotubes. The unchanged FABP4 level indicated no induction of transdifferentiation to adipocytes by this treatment. Reduced miR-100 in human mesenchymal cells [75] and subcutaneous [40,76] and visceral fat adipogenesis [40] is associated with adipocyte differentiation and fat accumulation, but miR-100 is upregulated in subcutaneous adipose tissue of obese subjects [76]. These observations suggest that miR-100 may play distinctive roles in adipogenesis and adipose tissue growth. In agreement, we observed higher number but smaller fat droplets in the agomiR-treated samples compared to NC. Similarly, miR-100 directly targets the promoter of adipogenesis, very low-density lipoprotein receptor, in human endothelial cells [77] and in pre-adipocytes [40]. Moreover, visceral adipose tissue of patients with non-alcoholic steatohepatitis (NASH) had lower miR-100 levels compared to patients without NASH [78], supporting that elevated miR-100 expression may play a role in fat accumulation and metabolism [79], and visceral fat content has a positive association with NASH development [80]. Whether miR-100 exerts these effects through *IGF1R* regulation needs further exploration.

Transgenic mice with miR-100 overexpression demonstrated reduced weight gain and increased glucose tolerance, insulin sensitivity, and energy expenditure compared to wild-type controls on a high-fat diet, despite similar calorie intake [81]. Moreover, serum and visceral adipose miR-100 levels were lower in obese normoglycemic and type 2 diabetic human individuals compared to lean and non-diabetics [40]. Above-mentioned studies have all reported a role for miR-100 in fat accumulation in adipose tissues and in adipogenesis, while our study demonstrates its involvement in skeletal muscle fat deposition, where it could regulate marbling. Future efforts are needed to test whether these findings could be translated to humans and whether miR-100 may play a causal role in insulin resistance. Hence, our results are in accordance with previous findings suggesting a causal role of miR-100 in fat accumulation and metabolism.

## Figures and Tables

**Table 1:** Oligonucleotide sequences for cloning.

Oligonucleotide	Sequence
**miR-100 WT-Sense**	5′pAAACTAGCGGCCGCTCCAGTAGAT**TACGGGTA**GTCAGTTGAC TCTAG-3′
**miR-100 WT-Antisense**	3′-pTTTGATCGCCGGCGAGGTCATCTA**ATGCCCAT**CAGTCAACTG AGATC-5′
**miR-100 Mutant-Sense**	3′pTTTGATCGCCGGCGAGGTCATCTA**TTTTTTTT**CAGTCAACTG AGATC-5′
**miR-100 Mutant-Antisense**	3′pTTTGATCGCCGGCGAGGTCATCTA**AAAAAAAA**CAGTCAACTG AGATC-5′

**Table 2:** Primer sequences for qPCR.

Gene	Accession Number	Forward Primer (5′)	Reverse Primer (3′)	Product Size, bp
*IGF1R*	NM_001244612.1	CCAAAACCGAAGCTGAGAAG	TCCGGGTCTGTGATGTTGTA	199
*MYOG*	NM_001111325.1	TGGGCGTGTAAGGTGTGTAA	TGCAGGCGCTCTATGTACTG	197
*MYOD*	NM_001040478.2	TTTGCCAGAGCAGGAGCCCCTC	TTCGAACACCTGAGCGAGCGC	123
*16S*	NC_006853.1	CTTGTATGAATGGCCGCACG	GATGTAGCGGGTCGTAGTGG	879
*HK2*	XM_015473383.2	CTCAGAGCGGCTCAAGACAA	GCACACCTCCTTGACGATGA	154
*COX7A2*	NM_175807.1	ACTGAGCCAAGATGCTACGG	TGAAGCCACAGCCAGTTCAT	242
*TFAM*	NM_001034016.2	GGCAGACTGGCAGGTATACAA	TGTGATGTGCCATCCCTAGC	225
*CPT1B*	NM_001034349.2	CTCTCCACTAGCCAGATCGC	CGCTGGGCATTTGTCTCTGA	197
*CD36*	NM_001278621.1	GACGGATGTACAGCGGTGAT	GGTTGCCAAGAGGTCTGGTT	250
*ACADM*	NM_001075235.1	CATGATCGCGCTGTTTAGGC	CAGGGTACTCGCCGGTTTTA	224
*FABP3* ^1^	NM_174313	GCGTTCTCTGTCGTCTTTCC	CTTGGTCATATTGCCCACCT	154
*FABP4* ^1^	NM_174314.2	GGATGGAAAATCAACCACCA	TGGACAACGTATCCAGCAGA	174
*ELOVL6*	NM_001102155.1	CATAGCACAGCCTCGGTCTA	TGCTGTGTCCTACCCCATTT	172
*ACC2*	NM_001205333.2	TCATTCACCTCCTGTCCACC	TAAACAGGAGTGAGCTGGGG	196
*GAPDH*	NM_001034034.2	GGGTCATCTCTGCACCT	ACAGTCTTCTGGGTGGCAGT	208

^1^ FABP3/4 sequences are from [54].

**Table 3:** Image analysis of Oil red O stained bovine satellite cell myotubes after treatment with oleic acid and agomiR or antagomiR compared to a negative control.

			Treatment				
	Negative Control		AgomiR		AntagomiR		*p*-Value
	Mean	SE	Mean	SE	Mean	SE	
Fat particles/mm²	1961.4 ^b^	196.0	2914.3 ^a^	163.0	2018.7 ^b^	30.2	0.007
Fat particle distance, µm	9.93 ^a^	0.37	8.89 ^b^	0.17	9.83 ^ab^	0.02	0.039
Fat area percentage	4.78 ^ab^	0.21	5.83 ^a^	0.54	3.99 ^b^	0.25	0.033
Fat particle size, µm²	24.45	1.55	19.71	0.65	19.57	1.16	0.043

^a,b^ different superscript letters indicate significant differences among treatments (*p* < 0.05).

## Data Availability

Data presented in this manuscript are available upon reasonable request to the corresponding author, with the exception of sensitivity data according to GDPR current regulations.
